# Body mass index, basal insulin and glycemic control in children with type 1 diabetes treated with the advanced hybrid closed loop system remain stable - 1-year prospective, observational, two-center study

**DOI:** 10.3389/fendo.2022.1036808

**Published:** 2022-10-11

**Authors:** Sebastian Seget, Przemysława Jarosz-Chobot, Agnieszka Ochab, Joanna Polanska, Ewa Rusak, Paulina Witoszek, Agata Chobot

**Affiliations:** ^1^ Department of Children’s Diabetology, Medical University of Silesia, Katowice, Poland; ^2^ Department of Pediatrics, Institute of Medical Sciences, University of Opole, Opole, Poland; ^3^ Department of Data Science and Engineering, Silesian University of Technology, Gliwice, Poland; ^4^ Department of Children’s Diabetology and Pediatrics, John Paul II Upper Silesian Child Health Centre, Katowice, Poland

**Keywords:** advanced hybrid closed-loop system, type 1 diabetes, children, body mass index, bmi, basal insulin, total daily insulin dose, time in range

## Abstract

**Background:**

Information on the influence of insulin treatment using advanced hybrid closed loop systems (AHCL) on body weight of young patients with type 1 diabetes (T1D) is scarce. The aim of this study was to observe whether there were any changes in body mass index (BMI) of children and adolescents with T1D treated using the Medtronic Minimed 780G AHCL after 1 year of follow up and to analyze potential associations between these changes and the insulin doses.

**Materials and methods:**

For 50 children and adolescents (age 5.4-16.8 years, 24 (48%) boys, T1D for 3.9 ± 2.56 years) using an AHCL system anthropometric and AHCL data were collected prospectively. BMI Z-scores and two-week AHCL records obtained after AHCL enrollment were compared with data after 6 months and also 1 year after starting AHCL.

**Results:**

The BMI Z-score of the patients at 1 year follow-up did not change from time of AHCL initiation (0.51 ± 2.79 vs 0.57 ± 2.85, p>0.05). There was a slight increase in total daily insulin per kg of body weight (0.67 ± 0.21 U/kg vs 0.80 ± 0.21 U/kg, p <0.001), but the percent of basal insulin was unchanged (34.88 ± 6.91% vs 35.08 ± 6.30%, p>0.05). We observed also no change (AHCL start vs after 1 year) in glycemic control parameters: average sensor glucose (131.36± 11.04 mg/dL vs 132.45 ± 13.42 mg/dL, p>0.05), coefficient of variation (34.99± 5.17% vs 34.06 ± 5.38%, p>0.05), glucose management indicator (6.45 ± 0.26% vs 6.48 ± 0.32%, p>0.05), and time spent in the range of 70–180 mg/dL (79.28 ± 8.12% vs 80.40 ± 8.25%, p>0.05).

**Conclusion:**

During the 1 year of follow-up the BMI of children and adolescents with T1D treated with an AHCL system remained stable. Although there was a slight increase in the total daily insulin dose, the percent of basal insulin was unchanged. The patients maintained recommended glycemic control.

## Introduction

The prevalence of overweight and obesity among youth with type 1 diabetes (T1D) is steadily increasing and reached even 35% in recent reports (1–4). Excessive body weight complicates attainment of recommended glucose control targets and is often tied with use of higher daily and basal insulin doses (5, 6). Obesity is also an independent, additional risk factor of macro- and microvascular complications, non-alcoholic fatty liver disease and polycystic ovary syndrome in individuals with T1D (7–13). Moreover youth with T1D and obesity are at higher risk of developing peripheral and cardiac autonomic neuropathy (14–16).

Evidence from the Diabetes Control and Complications Trial suggested that using high doses of insulin was related to weight gain in patients with diabetes (17). Although a later 10-year observation did not reach an identical conclusion (18) it seems that the relation between insulin treatment and excessive body weight or weight gain has not been fully explained (4, 8, 19–22).

Worth emphasizing is that children with T1D and obesity face many difficulties when attempting to treat both conditions. For example dieting and exercise, well known methods to decrease body weight, require additional education, selfcare and awareness due to the risk of hypoglycaemia (8, 23–25).

New technologies in the treatment of T1D, including the advanced hybrid closed loop (AHCL) system may perhaps be a good tool to make weight maintenance easier for children and adolescents with T1D (26). On the other hand these new technologies bring more flexibility in daily consumption. Knowing the action of insulin and that the mainstay of treatment for obesity are diet and exercise, one of the necessary approaches is developing treatment strategies with lowest possible daily insulin dose that would at the same not impair the glycemic control (8, 24, 25, 27).

Because of the increasing problem of overweight and obesity among children and adolescents with T1D it seems vital to further investigate this topic and increase the knowledge on how to prevent or treat excessive body weight in these patients. A recent meta-analysis showed no differences in weight gain in children treated with either insulin pumps or multiple daily injections (28, 29). However another study, that analyzed weight in children who switched from MDI to insulin pump, demonstrated different trends in weight gain. The results indicated a positive association between the basal insulin dose and rate of weight gain, while there was no association with the total daily insulin dose (30). Insulin pump treatment using recommended settings might help in reducing the basal insulin dose (31).

Considering the above aspects, our study aimed to observe if there were changes in weight of children and adolescents with T1D using an AHCL system after 1 year of follow up and to analyze potential associations with the daily and basal insulin doses.

## Materials and methods

### Patients

We enrolled for the study and followed prospectively 50 children and adolescents with T1D, treated with the AHCL system MiniMed 780G in automatic mode, at two regional pediatric diabetes centers (Department of Children’s Diabetology, University Clinical Hospital of the Medical University of Silesia in Katowice and Department of Pediatrics, University Clinical Hospital of the University of Opole, Poland), both Centers of Reference of the SWEET (Better control in Pediatric and Adolescent diabeteS: Working to crEate CEnTers of Reference) network. Diabetes care for children in Poland is centralized and carried out in regional centers belonging mostly to academic institutions. Inclusion criteria for the study were: age ≦̸ 18 years, as well as more than 70% of the sensor usage time, and more than 70% spent in automatic mode - to obtain reliable continuous glucose monitoring (CGM) and AHCL data.

### Methods

The study group was characterized by biometric parameters - age, sex, duration of T1D. Data from the AHCL system was automatically sent to the CareLink server and retrieved using CareLink Professional software (Medtronic MiniMed, USA). Two-week AHCL records as well as anthropometric parameters - body mass and height - were collected prospectively: right after AHCL enrollment, 6 months, and 1 year after starting AHCL. For each time point the body mass index (BMI) z-score was calculated using the individual’s weight and height and the World Health Organization (WHO) reference values (32). CGM readings were analyzed using GlyCulator 3.0 software (Medical University of Łódz, Poland) (33).

### Statistical analysis

The statistical analyses were performed using the Statistica 13.3 software (StatSoft, Inc., Tulsa, OK, USA). Descriptive statistics (mean, standard deviation, median, interquartile range, minimum and maximum values, coefficient of variation, and their 95% confidence intervals were calculated for each parameter. Data distribution was tested using the Shapiro–Wilk test. The differences between baseline and at 6 months follow-up as well as between baseline and at 1 year follow-up, were established, using Student’s t-test for dependent samples or the Wilcoxon signed rank test, whichever was appropriate according to the data distribution. Results were considered significant at p value lower than 0.05.

The study protocol was approved by the Local Bioethics Committee of the Medical University of Silesia in Katowice (Decision no. PCN/0022/KBI/83/2 of March 30, 2021).

## Results

The study included 50 children and adolescents with an average age of 9.9 ± 2.4 years (median: 9.7, range: 5.4-16.8), 24 (48%) of them were male. The average onset of T1D was 6.0 ± 2.9 (median: 5.7, range: 0.8-13.0) years and mean T1D duration was 3.9 ± 2.6 years (median: 3.63, range: 0.3-10.7).

**Figure 1:**
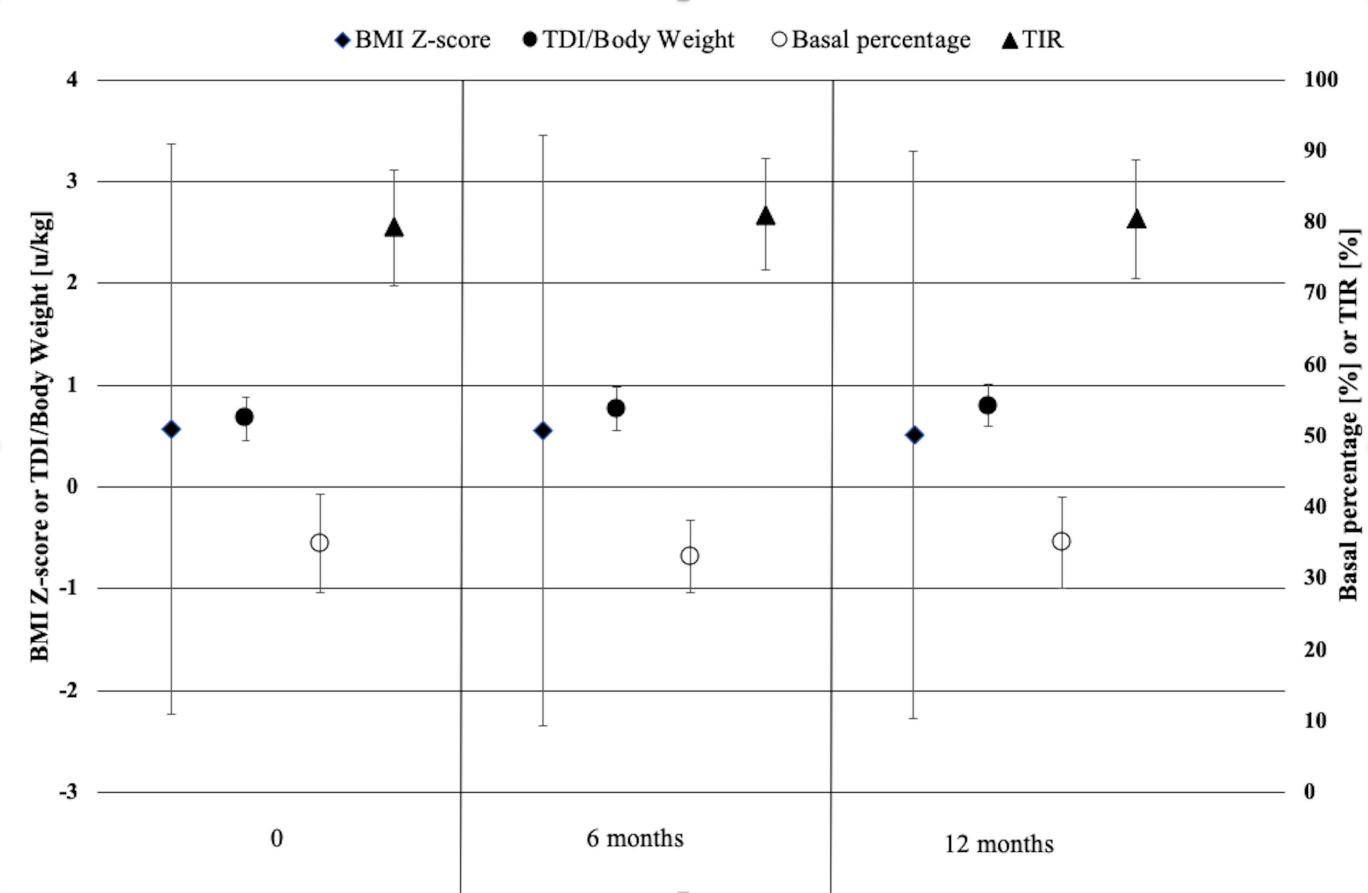
Body mass index, daily insulin doses, basal percentage and TIR of children and adolescents with type 1 diabetes using the advanced hybrid closed-loop system at initiation of the system, at 6 months and 1 year of follow-up.

BMI z-scores of the studied children and adolescents did not change significantly neither after 6 nor after 12 months of follow-up (**Table 1** and **Figure 1**). There was a slight increase in total daily insulin (TDI) dose from baseline (by 0.1 U/kg at 6 months and by 0.13 U/kg after 1 year of follow-up, p<0.001), however the percent of basal and bolus insulin remained stable. The amount of insulin in auto-corrective boluses increased significantly at 6 and 12 months (respectively by 0.82 U and 1.24 U, p<0.05) (**Table 1** and **Figure 1**).

**Table 1:** Body mass index, daily insulin doses and sensor data of children and adolescents with type 1 diabetes using the advanced hybrid closed-loop system at initiation of the system, at 6 months and 1 year of follow-up.

	AHCL First two weeks	AHCL Two weeks after six months	AHCL Two weeks after 1 year
BMI z-score	0.57 ± 2.85	0.55 ± 2.90	0.51 ± 2.79
Total daily insulin [U/kg]	0.67 ± 0.21	0.77 ± 0.21 *	0,80 ± 0.21 **
Basal insulin [%]	34.88 ± 6.91	33.16 ± 5.09	35.08 ± 6.36
Bolus insulin [%]	65.02 ± 6.9	66.84 ± 5.09	64.92 ± 6.36
Autocorrection [U]	2.85 ± 2.42	3.67 ± 2.64 *	4.09 ± 2.60 **
Sensor use [%]	95.1 ± 3.94	94.04 ± 4.69	93.8 ± 4.52
Smartguard [%]	92.32 ± 14.72	96.62 ± 5.70	97.56 ± 2.79 **
Average sensor glucose [mg/dl]	131.36± 11.04	132.46 ± 11.73	132.45 ± 13.42
CV [%]	34.99± 5.17	33.75 ± 5.02	34.06 ± 5.38
GMI [%]	6.45 ± 0.26	6.48 ± 0.28	6.48 ± 0.32
	Percent of sensor glucose values in range		
>250 mg/dl [%]	2.33 ± 2.52	2.34 ± 2.31	2.68 ± 3.48
180 - 250 mg/dl [%]	13.13 ± 5.74	12.83 ± 5.88	12.59 ± 5.78
70 - 180 mg/dl [%]	79.28 ± 8.12	81.16 ± 7.83	80.40 ± 8.25
54 - 70 mg/dl [%]	4.15 ± 2.70	2.95 ± 1.75 *	3.4 ± 2.34
<54 mg/dl [%]	1.11 ± 1,07	0.73 ± 0.77 *	0.93 ± 0.92

AHCL, Advanced Hybrid Closed-Loop System; U, unit; CV, Coefficient of variation; GMI, Glucose Management Indicator; *, significant difference (p<0.05) between baseline 2 weeks using AHCL and the 2 weeks after 6 months, ** significant difference (p<0.05) between baseline 2 weeks using AHCL and the 2 weeks after 1 year follow-up.

In general we did not observe changes after 6 or after 12 months of follow-up in glycemic control parameters: average sensor glucose, coefficient of variation, glucose management indicator, and percent of sensor glucose values in different ranges. Only the percent of time spent in range 54-70 mg/dl and <54 mg/dl was significantly reduced after 6 months (p<0.05), but did not differ from baseline after one year of treatment (**Table 1**). After one year, the use of the AHCL auto mode (% of time using Smartguard) increased significantly (p<0.05), while maintaining similar time of sensor use (**Table 1**).

## Discussion

We describe stable BMI z-scores of children and adolescents with T1D using the AHCL system during a 1-year follow up which is the first such long observation (34--45). Similar results, also for a pediatric cohort of individuals with T1D, were obtained by Tornese et al., who reported no change in BMI z-score after 6 months of insulin treatment using either the hybrid closed-loop (Minimed 670G) or AHCL (Minimed 780G) systems. Noteworthy, our patients were characterized by more optimal glycemic control (lower GMI 6.48% vs 7.1% and higher TIR 81.16% vs 72%) after 6 months of AHCL use with a similar time spent in auto-mode (96.62% vs 96) and percent of sensor use (94.04% vs 92%) (36). Among the growing number of studies evaluating the AHCL Minimed 780G system these are to our knowledge the only two investigating the associations with BMI z-score (34–45). Former studies that observed BMI in children with T1D using insulin pumps other than AHCL showed not unequivocal results although the latest metaanalysis suggested no change in body mass (28, 29).

Weight gain was linked to the basal insulin dose and seemed to be independent from the TDI dose (30). Our observations from this study stay in line with the previous findings - no change in BMI z-score was accompanied by a slight increase in TDI. The percentage of basal insulin was 34.88% and did not change after 6 or 12 months of AHCL use. Also the largest pediatric AHCL study during which 790 patients 15 years of age were followed for 6 months (time in auto mode at 6 months 94.9%) revealed an increase in TDI (44). In two other investigations the transition from sensor augmented pump with low glucose suspend system (SAP-LGS) or predictive low glucose suspend system (SAP-PLGS) to AHCL - contrary to our findings - was associated with a decrease in basal insulin with a simultaneous increase in bolus insulin, which is most likely due to self-correction (39, 45). This small but significant increase of TDI in our cohort may be partially explained by the fact that the studied population was younger and some of these children might have entered puberty during the observation time.

Another interesting observation from this study is the increased use of the AHCL auto mode and no change of time of sensor use after one year of observation. The high and unchanged sensor use that was found also in another study may result from the necessity to use it to operate the Minimed system in automatic mode (36). The increasing time in auto-mode is optimistic and would suggest that the patients are not only keen to use it as a novelty but with time learn to rely on it and use it more.

The studied cohort showed a high TIR and other sensor parameters very well fitting the recommendations. This optimal control was maintained during the 1 year of follow-up. Authors of shorter, up to 6 months, observations described significant improvements in glycemic control parameters after switching to AHCL from SAP-LGS/PLGS. However the cohort presented in this study as well as the subgroup of patients from Poland that were included in the publication by Arrieta et al. had the best baseline glycemic control (44).

The above discussed aspects suggest that the AHCL may help to sustain good glycemic control without a risk of increasing body weight. The unchanged percent of time <70 and <54 mg/dl can be also interpreted as no increase in severe hypoglycaemia risk. If AHCL could be a beneficial tool for overweight or obese children with T1D in terms facilitaing weight reduction without impairment in glycemic control requires further studies.

The novelty of this study is the longest, 1 year follow-up of the AHCL use in auto-mode in the pediatric population combined with the assessment of BMI z-score changes. Another strength of this investigation is the youngest observed until now (mean age: 9.88 ± 2.44) (34–45) cohort characterized by good glycemic control parameters. Nevertheless we acknowledge the limitations, which include the lack of the assessment of the amount of carbohydrates consumed as well as no detailed dietary evaluation at baseline and follow up that would allow us to note any changes in eating behaviors.

## Conclusion

During the 1 year of follow-up the BMI z-score of children and adolescents with T1D using an AHCL system remained stable. Although there was a slight increase in the total daily insulin dose, the percent of basal insulin was unchanged. The patients maintained recommended glycemic control.

## Data availability statement

The datasets generated and analyzed for this study are available from the corresponding or first author upon reasonable request.

## Ethics statement

The studies involving human participants were reviewed and approved by Local Bioethics Committee of the Medical University of Silesia in Katowice (Decision no. PCN/0022/KBI/83/2 of March 30, 2021). Written informed consent to participate in this study was provided by the participants’ legal guardian/next of kin.

## Author contributions

SS researched data, performed statistical analysis, participated in data interpretation and drafting the manuscript. PJ-C designed the study and reviewed and edited the manuscript. AO, ER, and PW participated in researching data and contributed to the drafting of the manuscript. JP supervised the statistical analysis and participated in data interpretation. AC reviewed the study design, participated in data interpretation, reviewed and edited the manuscript. All authors approved the final version of the manuscript. AC and SS are the guarantors of this work and, as such, had full access to all the data in the study and take responsibility for the integrity of the data and the accuracy of the data analysis.

## Funding

JP was financed by SUT grant no 02/070/BK_22/0033 project.

## Conflict of interest

P-JC has received speaker honoraria from Medtronic, DexCom, Abbott, Ypsomed, and Roche, was a member of the advisory boards for Medtronic and Abbott and received research support from Medtronic. SS has received speaker honoraria from Medtronic and Ypsomed.

The remaining authors declare that the research was conducted in the absence of any commercial or financial relationships that could be construed as a potential conflict of interest.

## Publisher’s note

All claims expressed in this article are solely those of the authors and do not necessarily represent those of their affiliated organizations, or those of the publisher, the editors and the reviewers. Any product that may be evaluated in this article, or claim that may be made by its manufacturer, is not guaranteed or endorsed by the publisher.
